# Immunological Status of Bladder Cancer Patients Based on Urine Leukocyte Composition at Radical Cystectomy

**DOI:** 10.3390/biomedicines9091125

**Published:** 2021-08-31

**Authors:** Elisabet Cantó, Óscar Rodríguez Faba, Carlos Zamora, Maria Mulet, Maria Soledad Garcia-Cuerva, Ana Palomino, Georgia Anguera, Alberto Breda, Pablo Maroto, Sílvia Vidal

**Affiliations:** 1 Inflammatory Diseases, Institut de Recerca de l’Hospital de la Santa Creu i Sant Pau, Biomedical Research Institute Sant Pau (IIB Sant Pau), 08041 Barcelona, Spain; ecanto@santpau.cat (E.C.); CZamora@santpau.cat (C.Z.); MMulet@santpau.cat (M.M.); 2 Department of Urology, Fundació Puigvert, Autonomous University of Barcelona, 08025 Barcelona, Spain; ORODRIGUEZ@fundacio-puigvert.es (Ó.R.F.); apalomino@fundacio-puigvert.es (A.P.); albbred@hotmail.com (A.B.); 3 Department of Pathological Anatomy, Fundació Puigvert, 08025 Barcelona, Spain; msgarciac@fundacio-puigvert.es; 4 Department of Medical Oncology, Hospital de la Santa Creu i Sant Pau, Autonomous University of Barcelona, 08025 Barcelona, Spain; GAnguera@santpau.cat (G.A.); JMaroto@santpau.cat (P.M.)

**Keywords:** MIBC, urine, leukocytes, PD-L1, bladder

## Abstract

Background: Bladder cancer (BC) is the ninth most common malignancy worldwide, with high rates of recurrence. The use of urine leukocyte composition at the time of radical cystectomy (RC) as a marker for the study of patients’ immunological status and to predict the recurrence of muscle-invasive bladder cancer (MIBC) has received little attention. Methods: Urine and matched peripheral blood samples were collected from 24 MIBC patients at the time of RC. Leukocyte composition and expression of PD-L1 and PD-1 in each subpopulation were determined by flow cytometry. Results: All MIBC patients had leukocytes in urine. There were different proportions of leukocyte subpopulations. The expression of PD-L1 and PD-1 on each subpopulation differed between patients. Neoadjuvant chemotherapy (NAC), smoking status, and the affectation of lymph nodes influenced urine composition. We observed a link between leukocytes in urine and blood circulation. Recurrent patients without NAC and with no affectation of lymph nodes had a higher proportion of lymphocytes, macrophages, and PD-L1+ neutrophils in urine than non-recurrent patients. Conclusions: Urine leukocyte composition may be a useful tool for analyzing the immunological status of MIBC patients. Urine cellular composition allowed us to identify a new subgroup of LN− patients with a higher risk of recurrence.

## 1. Introduction

Bladder cancer (BC), the ninth most common malignancy worldwide [1], has high rates of recurrence and progression [2]. For MIBC, European guidelines recommend NAC plus RC with pelvic lymphadenectomy as the primary treatment of choice [3] when attempting local disease control. Lymph node dissection is an essential step in the treatment of MIBC because it is known that approximately 25–30% of patients have lymph node metastasis at the time of surgery [4,5], and also because lymph node status is one of the most important indicators of long-term overall survival (OS) and recurrence-free survival (RFS). However, approximately 20–40% of patients with organ-confined and negative lymph nodes (LN−) recur within the first two to three years [5].

The risk factors for bladder cancer include family history, history of bladder infections, occupational exposure to aromatic amines and polycyclic aromatic hydrocarbons, and cigarette smoking [6]. Tobacco use is one of the primary and most common risk factors for BC. The precise mechanism of BC induction through the use of cigarettes, although unknown, seems to be related to the chemicals found in cigarettes [7].

In recent years, there has been a widespread effort to identify biomarkers in urine and blood to diagnose bladder cancer and to determine the outcome of patients. Numerous urinary tests have been developed to avoid unnecessary cystoscopies. None of these markers have been accepted for diagnosis and follow-up in clinical practice [8,9]. Currently, promising novel urinary biomarkers, assessing multiple targets, have been tested in prospective multicenter studies, with a very high negative predictive value [10]. The concentrations of urine- and plasma-soluble proteins, such as VEGF, endostatin, stress proteins, and cytokines, are higher in bladder cancer patients than healthy controls and are useful for the diagnosis and staging of bladder cancer [11]. The presence of leukocyte cells in urine after Bacillus Calmette–Guerin (BCG) administration seems to be a surrogate urine biomarker of immune system activation [12]. In addition, an elevated neutrophil-to-lymphocyte ratio (NLR) in the blood of MIBC patients has been associated with a decrease in their response to neoadjuvant chemotherapy and a shorter disease-specific and OS [13]. It has also been shown that higher tumor-infiltrating neutrophil counts and NLR predict both an advanced pathological stage and poorer survival, confirming the link between circulation and the tumor milieu [13]. For example, blood natural killer cell biomarkers [14] and CD63+ expression on blood neutrophils have been used to stratify the immunological risk for patients with different cancers [15,16].

There have been few studies investigating the use of non-invasive markers in urine for predicting the outcome of bladder cancer patients. To our knowledge, only Wong et al. have described that a higher count/mL of lymphocytes in urine and PD-1 high in urine CD3 + CD8+ in MIBC patients were significantly associated with disease recurrence [17]. However, the influence of treatment and risk factors on urine cell composition has not been properly investigated. To this end, we therefore analyzed the influence of NAC, smoking status, and lymph node affectation at the time of RC on urine cell composition and PD-L1 and PD-1 expression. We observed that all MIBC patients had leukocytes in their urine, indicating the activation of the immune system [12]. However, the proportion of the leukocyte subpopulations differed between patients, suggesting that MIBC patients did not have the same immunological status at the moment of RC. In addition, we also measured the expression of PD-L1 and PD-1 on each leukocyte subpopulation, including neutrophils. Determining urine cellular composition allowed us to identify a new subgroup of LN− patients at a higher risk of recurrence.

## 2. Material and Methods

### 2.1. Patients

Urine and peripheral blood samples were collected from 24 patients with muscle-invasive bladder cancer (MIBC) undergoing radical cystectomy (RC) and from 4 healthy donors (HD). Pathological reports were provided by a single uropathologist following the European Guidelines for the assessment of tumor specimens [18]. Our cohort included patients undergoing neoadjuvant chemotherapy (*n* = 9) and patients not undergoing it (*n* = 15). Infection was an exclusion criterion. Patients were followed up for 22.50 ± 9.42 months. Written informed consent was obtained from all the participants in the study and ethical approval was granted by the Puigvert Hospital Institutional Ethics Committee (2016/05c, 27 June 2016).

### 2.2. Isolation of Urine Cells

The median volume of morning urine before RC from MIBC patients and HD was 57 mL (range, 6–100 mL). Urine samples were processed within 1 h, as described previously [19], to reduce dead cells; filtered by gauze; and pulled down by centrifugation at 800× *g* for 10 min at room temperature. Supernatant was collected and stored at −20 °C. The cell pellet was resuspended and washed with PBS and 0.5% BSA (bovine serum albumin, Calbiochem, Madrid, Spain). Red blood cells were lysed using RBC lysis buffer (BioLegend, San Diego, CA, USA) for five minutes at room temperature. The pellet was washed twice with PBS and 0.5% BSA and total events were counted by flow cytometry (MACSQuant Analyzer 10 flow cytometer, Miltenyi Biotec, Bergisch Gladbach, Cologne, Germany).

### 2.3. Staining Urine Cells

A total of 10 × 10^6^ events were stained using a panel of antibodies. All urines were analyzed by flow cytometry without antibodies to allow us to draw the limit of detection, excluding the autofluorescence. To avoid urine autofluorescence, we could not use fluorochromes detected by 450–630 nm channels. Viable cells were gated based on dye fluorescent (LIVE/DEAD TM Fixable Violet Dead Cell Stain Kit, Thermo Fisher Scientific, Waltham, MA, USA) and all the analyses were done considering only viable cells. Doublets were excluded using the FSC parameter. To detect leukocyte subpopulations and their expression of PD-L1 and PD-1, the pellet was stained using CD45-APC-Vio770 (Miltenyi Biotec), CD14-APC (Immunotools, Gladiolenwg, Friesoythe, Germany), CD16-PercpVio700 (Myltenyi Biotec), CD3-PeCy5, CD8-PCy7, and PD-L1-APC and PD-1-PE (BioLegend). We used anti-CD3 and -CD8 antibodies to discriminate between CD4+ (CD3 + CD8−), CD8+ (CD3 + CD8+), CD3− CD8+, and CD3− CD8−. The expression of PD-L1 and PD-1 was determined on all cell subpopulations. Lymphocyte subpopulations and the expression of PD-L1 and PD-1 on each subpopulation were only determined in the MIBC urines with >1 % of lymphocytes to avoid possible artifacts (*n* = 18). To detect epithelial tumor cells and the expression of PD-L1, the cellular pellet was stained with EpCAM-PE-Vio770 (CD326; Miltenyi Biotec) and PD-L1-APC (BioLegend). The expression of PD-L1 and PD-1 was determined based on the limit of detection delimitated by the fluorescence minus one (FMO). Samples were acquired and analyzed with the MACSQuant Analyzer 10 flow cytometer (Miltenyi Biotec).

### 2.4. Peripheral Blood

Peripheral blood samples were collected from matched patients with MIBC before RC and processed for the analysis of leukocytes. Briefly, 100 µL of blood were washed with 2 mL of PBS. The pellet was stained with a panel of antibodies to analyze leukocyte subpopulations: CD45 Vioblue (Miltenyi Biotec), CD3-PeCy5, CD8-PCy7, and PD-L1-APC and PD-1-PE (BioLegend). We used CD3 and CD8 antibodies to discriminate between CD4+ (CD3 + CD8−), CD8+ (CD3 + CD8+), CD3− CD8+, and CD3− CD8−. The expression of PD-L1 and PD-1 was determined in all cell population. The expression of PD-L1 and PD-1 was determined based on the limit of detection delimitated by FMO. Samples were acquired and analyzed with the MACSQuant Analyzer 10 flow cytometer (Miltenyi Biotec).

### 2.5. Histology

Bladder biopsies were frozen in liquid Nitrogen and kept at −80 °C until used. Five micrometers sections were cut at different levels and stained with hematoxylin and eosin. Slides were examined without prior knowledge of the urine leukocyte population. The mean of the area analyzed in all biopsies was 2.5 cm^2^. Based on the morphology and different staining of cell nuclei (HE), lymphocytes were counted in all biopsy sections three times by two different observers, and the values were shown as a range (0–100, 100–1000, and >1000 cells). The average of the repeat counts was used for the statistical analyses [20].

### 2.6. Urine IL-6 and IL-8 Concentration

The concentration of IL-6 (Immunotools) and IL-8 (Mabtech, Augustendalstorget, SE-131 28 Nacka Strand, Sweden) was determined by the ELISA manufacturer’s instructions. The limits of concentrations were 6 pg/mL for IL-6 and 4 pg/mL for IL-8. Urine cytokines were normalized with the content of urine creatinine measured using an Elisa Kit (R & D, McKinley Place NE, Minneapolis, MN, USA). Results are expressed as the concentration of cytokine per milligram of creatinine.

### 2.7. Statistical Analysis

Statistical analyses were performed using GraphPad Prism 7 (San Diego, CA, USA). The Kolmogorov–Smirnov test was applied to test the normal distribution of the data. Continuous variables were presented as median and interquartile range (25th–75th percentile IQR). The Mann–Whitney test was used for the comparison of independent variables. The chi-square test was used for the comparison of frequencies and correlations were analyzed with Spearman coefficients. *p*-values of less than 0.05 were considered significant.

## 3. Results

### 3.1. Demographic Characteristics of Muscle-Invasive Bladder Cancer (MIBC) Patients and Urine Cell Composition

In Table 1, we show the demographic and clinical characteristics of 24 patients diagnosed with MIBC at the time of RC.

In Table 2 and Supplementary Figure S1, we show the urine cellular composition based on the presence of leukocyte and epithelial cells, the expression of PD-L1 and PD-1 on each cell population, and the absolute number per mL of urine and the percentage of viable CD45+ and CD326+ cells. The viability of CD45+ cells ranged between 97.4% and 100%. The viability of CD326+ cells in 22 samples was >80%, and in only two samples was it <60%. The urine CD45+/mL and lymphocytes/CD326+ ratios correlated positively with the normalized urine concentration of IL-8 (3.95 ± 6.17 pg/mg of creatinine; *r* = 0.782, *p* < 0.001, and *r* = 0.630, *p* = 0.009, respectively) and IL-6 (4.01 ± 14.5 pg/mg of creatinine; *r* = 0.781, *p* < 0.001, and *r* = 0.650, *p* = 0.05, respectively).

**Figure 1:**
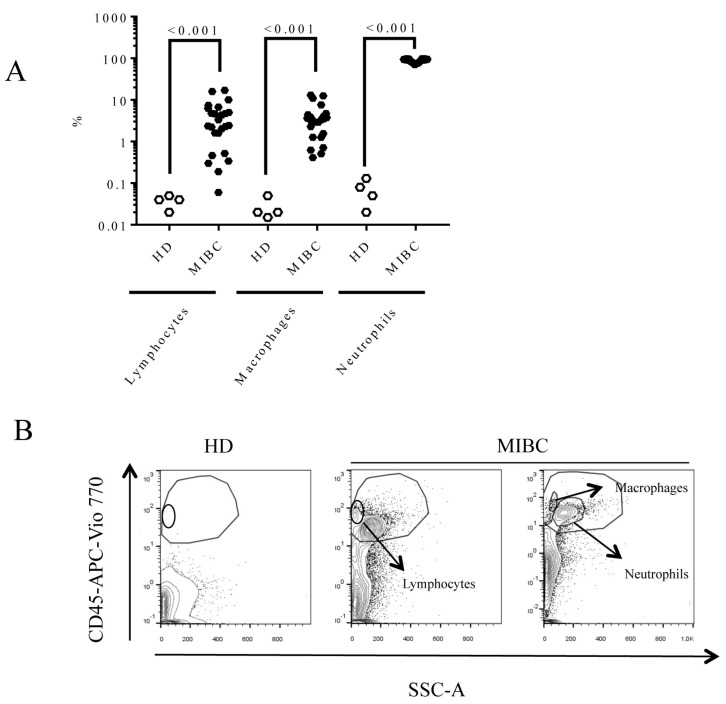
Urine cell composition from MIBC patients and HD. Urine was processed and CD45+ population was determined by flow cytometry as described in Materials and Methods. The percentage of lymphocytes, macrophages, and neutrophils was determined within the CD45+ population. (**A**) Percentage of lymphocytes, macrophages, and neutrophils present in the urine of HD (*n* = 4) and MIBC patients (*n* = 24); (**B**) dot plots of a flow cytometry example of urine from HD and urine samples from MIBC patients, showing the position of lymphocyte, macrophage, and neutrophil populations. The Mann–Whitney test was used for the comparison of independent variables.

We were able to detect lymphocytes, macrophages, and neutrophils in the urine of MIBC patients. However, not all patients had the same proportion of lymphocytes in their urine, as demonstrated in Figure 1A.

In the urine from healthy donors (HD), the percentage of lymphocytes in the leukocyte gate (CD45+) was 0.04 (IQR: 0.025–0.047). In contrast, the urine from MIBC patients contained more lymphocytes (2.42 (IQR: 1.06–5.625), *p* < 0.001), macrophages (3.35 (1.34–4.27), *p* < 0.001), and neutrophils (90.75 (80.86–94.95), *p* < 0.001). In Figure 1B, we show representative dot plots of cell composition from HD and two different MIBC patients. In Supplementary Figures S2 and S3, we show the gating hierarchy for CD45+ subpopulations and CD326+ cells from urine samples and blood and the expression of PD-1 and PD-L1 in each leukocyte subpopulation.

### 3.2. Neoadjuvant Chemotherapy Influences Urine Composition in MIBC Patients

**Figure 2:**
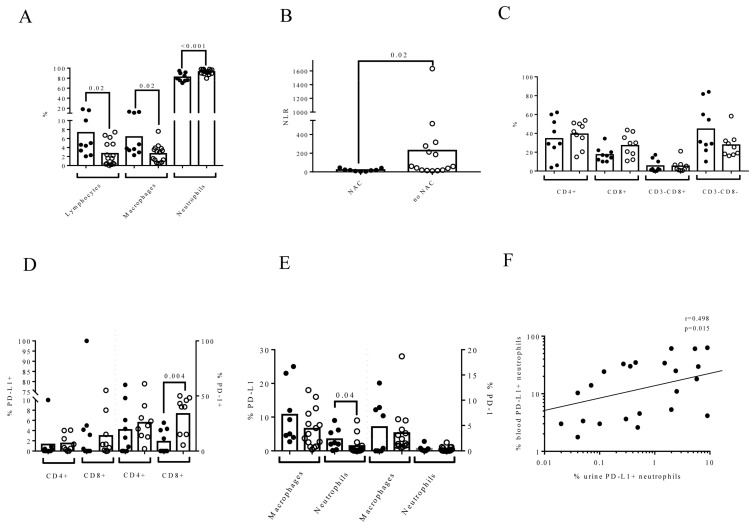
Neoadjuvant chemotherapy (NAC) before RC influenced urine cell composition. Flow cytometry was conducted to determine the cell composition and expression of PD-L1 and PD-1 on each urine cell subpopulation. Solid circles represent patients who received neoadjuvant chemotherapy (NAC) before RC and open circles represent patients who did not receive NAC. The phenotype of the lymphocytes was determined in MIBC patients with >1% of lymphocytes (*n* = 18) to avoid possible artifacts. (**A**) The effect of NAC on the percentage of urine lymphocytes, macrophages, and neutrophils gated inside the leukocyte population; (**B**) the urine NLR; (**C**) the phenotype of lymphocytes in the urine based on the expression of CD3 and CD8 (*n* = 18); (**D**) the expression of PD-L1 and PD-1 on CD4+ and CD8+ urine lymphocytes (*n* = 18); (**E**) the expression of PD-L1 and PD-1 on macrophages and neutrophils in urine samples from MIBC patients (*n* = 24); and (**F**) correlation between the percentage of PD-L1+ neutrophils from urine and matched blood. The Mann–Whitney test was used for the comparison of independent variables. Spearman’s coefficient was used to correlate independent variables.

We observed that patients who received neoadjuvant chemotherapy (NAC) had a different urine cell composition than patients who did not receive it. Namely, patients who underwent NAC had a higher percentage of lymphocytes than patients who did not receive NAC (4.61 (IQR: 2.81–12.99) vs. 1.61 (IQR: 0.34–4.76), *p* = 0.029) (Figure 2A). However, we did not find any association between the effectiveness of NAC and urine composition (data not shown).

There were also differences in the percentages of macrophages (*p* = 0.022) and neutrophils (*p* < 0.001), and, as a result, the NLR was higher in patients who received NAC therapy than those who did not receive it (*p* = 0.021) (Figure 2B). NAC did not modify the proportion of urine lymphocyte subpopulations (Figure 2C), though it decreased the percentage of urine PD-1+ CD8+ lymphocytes (0 (IQR: 0–19.45) vs. 40 (IQR:15–47.15), *p* = 0.004) (Figure 2D) and it increased the percentage of urine PD-L1+ neutrophils (2.2 (IQR: 0.77–5.95) vs. 0.3 (IQR: 0.05–1.51), *p* = 0.041) (Figure 2E). Interestingly, we found a positive correlation between the percentages of PD-L1+ neutrophils in urine and matched blood samples from MIBC patients (*r* = 0.498, *p* = 0.015) (Figure 2F).

### 3.3. Smoking Status Influences Urine Composition in MIBC Patients

**Figure 3:**
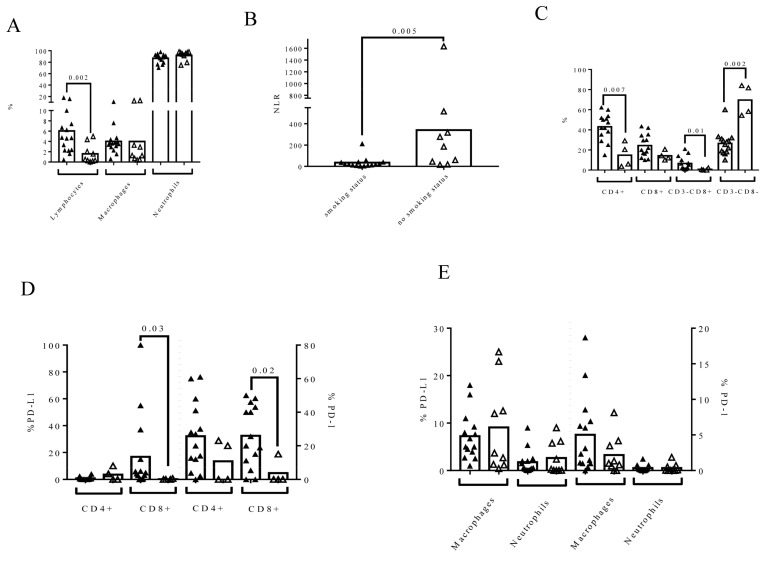
Smoking status influenced urine cell composition at the time of RC. Flow cytometry was conducted to determine the cell composition and expression of PD-L1 and PD-1 on each urine cell subpopulation. Filled triangles represent patients who smoked and open triangles represent patients who did not smoke. The phenotype of lymphocyte subpopulations was determined in MIBC patients with >1% of lymphocytes (*n* = 18) to avoid possible artifacts. (**A**) The effect of smoking status on the percentage of urine lymphocytes, macrophages, and neutrophils gated inside leukocyte population; (**B**) the urine NLR; (**C**) the phenotype of urine lymphocytes based on the expression of CD3 and CD8 (*n* = 18); (**D**) the expression of PD-L1 and PD-1 on CD4+ and CD8+ urine lymphocytes (*n* = 18); and (**E**) the expression of PD-L1 and PD-1 on macrophages and neutrophils in the urine of MIBC patients (*n* = 24). The Mann–Whitney test was used for the comparison of independent variables.

Patients who were smokers had a higher percentage of urine lymphocytes than non-smoking patients (4.69 (IQR: 2.3–7.42) vs. 0.52 (IQR: 0.24–3.21), *p* = 0.002) (Figure 3A). Smoking status did not affect the percentage of macrophages or neutrophils in urine. However, the NLR was also significantly different in both groups of patients (*p* = 0.005) (Figure 3B). Smoking status also affected the percentage of lymphocyte subpopulations in urine. An increase in T CD4+ (*p* = 0.007) and CD3− CD8+ cells (*p* = 0.016) and a decrease in CD3− CD8- cells (*p* = 0.002) were observed in smokers (Figure 3C). The expression of PD-L1 and PD-1 was also affected by smoking status (Figure 3D). Smoking patients had higher percentages of PD-L1 + CD8+ cells and PD-1 + CD8+ cells than patients who did not smoke: PD-L1+ CD8+ (4.58 (IQR: 0.31–20.5) vs. 0 (IQR: 0–0.5), *p* = 0.035) and PD-1+ CD8+ (22.83 (IQR: 9.72–43.75) vs. 0 (IQR: 0–11.25), *p* = 0.028). The expression of PD-L1 and PD-1 on macrophages or neutrophils was not affected by smoking status (Figure 3E).

### 3.4. The Affectation of Lymph Nodes at RC Time Influences Urine Cell Composition

**Figure 4:**
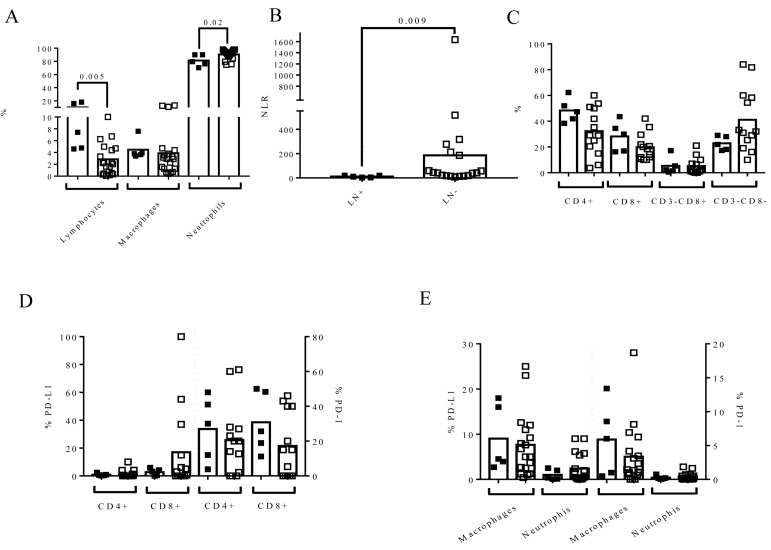
Lymph node positivity (LN+) at the time of RC influenced urine cell composition. Urine samples from MIBC patients (*n* = 24) were processed as described in Materials and Methods. Flow cytometry was conducted to determine the cell composition and expression of PD-L1 and PD-1 on each urine cell subpopulation. Black squares represent patients with LN positive at the time of RC and open squares represent negative patients. The phenotype of the lymphocyte subpopulation was determined in MIBC patients with >1% of lymphocytes (*n* = 18) to avoid possible artifacts. (**A**) The effect of LN positivity (LN+) on the percentage of urine lymphocytes, macrophages, and neutrophils gated inside the leukocyte population; (**B**) the urine NLR; (**C**) the phenotype of urine lymphocytes based on the expression of CD3 and CD8 (*n* = 18); (**D**) the expression of PD-L1 and PD-1 on CD4+ and CD8+ urine lymphocytes (*n* = 18); and (**E**) the expression of PD-L1 and PD-1 on macrophages and neutrophils in the urine of MIBC patients (*n* = 24). The Mann–Whitney test was used for the comparison of independent variables.

The urine of patients with positive lymph nodes (LN+) had a higher percentage of lymphocytes (LN+7.42 (IQR: 4.68–16.99 vs. LN-2.23 (IQR: 0.46–4.69) *p* = 0.005) and a lower percentage of neutrophils (LN+ (79.65 (IQR: 73.34–90) vs. LN- (93 (IQR: 87.29–96.68), *p* = 0.021) than the urine of patients without affectation (Figure 4A).

As a result, the NLR was also different in the two groups of patients (*p* = 0.009) (Figure 4B). Patients with the highest NLR in Figure 2B, Figure 3B and Figure 4B were the same. No differences in urine PD-L1+ and PD-1+ lymphocytes were observed according to the affectation or not of lymph nodes (Figure 4C–E).

### 3.5. Negative Correlation between Lymphocytes in Urine and in Bladder Biopsy at the Time of RC

**Figure 5:**
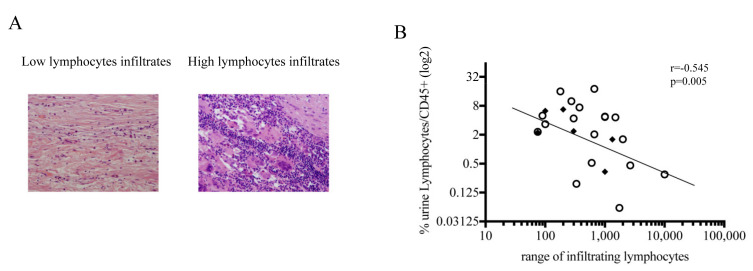
Relationship between urine lymphocytes at RC time and infiltrate lymphocytes present in the RC biopsy specimen. (**A**) Example of biopsies obtained at the time of RC from MIBC patients showing the lack infiltrating lymphocytes (20×), and (**B**) correlation between infiltrate lymphocytes in biopsies and the percentage of lymphocytes present in the urine of matched MIBC patients. Black diamonds correspond to patients who suffered recurrence. Spearman’s coefficient was used to correlate independent variables.

The number of lymphocytes present in the biopsies was calculated on hematoxylin-eosin specimens with two different supervisors as described in Material and Methods. Bladder biopsies from two representative patients (one with a low number and one with a high number of infiltrating lymphocytes) are shown in Figure 5A. The approximate number of infiltrating lymphocytes in bladder biopsies negatively correlated with the percentage of urine lymphocytes (*r* = −0.545, *p* = 0.005) (Figure 5B).

### 3.6. The Composition of Urine at the Time of RC Time Is Associated with the Recurrence of LN (−) in Patients

**Figure 6:**
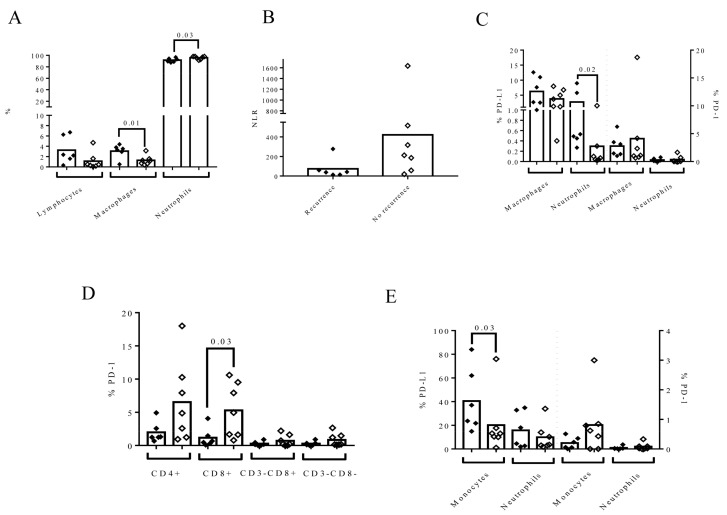
Urine and blood cell composition at the time of RC could predict recurrence in those patients who had not received neoadjuvant chemotherapy and were LN negative. At the time of RC, a different urine cell composition was observed between patients who recurred (black diamond) and patients who had not recurred (open diamond) during follow-up. The phenotype of lymphocyte subpopulation was not determined in MIBC patients since five patients had <1% of urine lymphocytes. (**A**) Percentage of urine lymphocytes, macrophages, and neutrophils gated inside the leukocyte population (*n* = 13); (**B**) the urine NLR; (**C**) the expression of PD-L1 and PD-1 on macrophages and neutrophils in the urine of MIBC patients (*n* = 13); (**D**) the phenotype of blood lymphocytes based on the expression of CD3 and CD8 (*n* = 13); and (**E**) the expression of PD-L1 and PD-1 on macrophages and neutrophils in the blood of MIBC patients (*n* = 13). The Mann–Whitney test was used for the comparison of independent variables.

To analyze the association between urine cellular composition and recurrence during follow-up, we selected patients with LN− at the time of RC who did not receive NAC (Figure 6 and Supplementary Table S1).

The intention behind this approach was to discard the effect of chemotherapy on the analysis of urine composition. When we stratified these patients based on their recurrences, no differences in the months of follow-up were observed (23 ± 9 vs. 29 ± 5, *p* = *n*.s.). Patients who suffered a recurrence during follow-up tended to have more urine lymphocytes (*p* = 0.07), a higher percentage of macrophages (*p* = 0.016), and a lower percentage of neutrophils (*p* = 0.038) in their urine than those without recurrence (Figure 6A). As a result, the NLR in urine tended to be lower in patients with recurrence (*p* = 0.07) (Figure 6B). In addition, the percentage of urine PD-L1+ neutrophils was higher in patients who recurred than patients who did not (0.51 (IQR: 0.405–6.57) vs. 0.07 (IQR: 0.04–0.3), *p* = 0.021), as shown in Figure 6C. The approximate number of infiltrating lymphocytes in bladder biopsies of patients who suffered recurrence in this subgroup of patients was negatively correlated with the percentage of urine lymphocytes (*r* = −0.586, *p* = 0.038, Figure 5B). This subgroup of patients with T3–T4 stage had a tendency to have higher percentage of CD326+ CD45− (1.96 (0.35–8.92)) than patients with T1 (0.02 (0.02–0.02)), T0 (0.49 (0.31–1.1)), or pTis (0.61 (0.61–0.61)), but without statistical significance. Furthermore, in patients with recurrence, blood NLR also tended to be higher than in those without recurrence (*p* = 0.051). We also found that the percentage of PD-1+ CD8+ in the blood of patients with recurrence was lower (*p* = 0.038) (Figure 6D) and the percentage of PD-L1+ monocytes was higher (*p* = 0.035) (Figure 6E). We also analyzed whether smoking status affected these results, but we found that the proportion of smoking patients in each recurrence group was not different (*p* = 0.592).

## 4. Discussion

The presence of lymphocytes, macrophages, and neutrophils in urine from MIBC patients suggests the involvement of the immune system in the pathology of MIBC. In our cohort, urine leukocyte composition was associated with NAC, smoking status, and affectation of LN at the time of RC. Interestingly, by following our strategy, we were able to identify patients with no lymph node affectation who recurred during follow-up.

MIBC patients who received NAC showed a higher percentage of urine lymphocytes and macrophages but a lower percentage of neutrophils than patients who did not undergo this therapy. Although there is no information regarding the influence of cancer treatments on urine leukocytes, several reports describe the influence of treatments on the leukocyte infiltration of biopsies. Recently, Seiler et al. described significant changes in the biopsies of NAC-treated MIBC patients based on transcriptome-wide gene expression and IHC [21]. The influence of NAC on neutrophil infiltrates has been demonstrated in benign lymph node biopsies from MIBC patients [22]. Neutrophil infiltration is also evident after BCG therapy [23]. The role of these infiltrating neutrophils is more controversial. In bladder cancer and other types of solid tumors, they seem to be essential for the anti-tumor effect [23,24]. However, in gastric cancer patients, PD-L1+ neutrophils were shown to suppress T-cell function and promote disease progression [25]. In our cohort, we did not find any association between urine PD-L1+ neutrophils and disease progression.

Our results show that patients who received NAC had a lower PD-1+ expression on CD8+ but a higher PD-L1+ expression on neutrophils than those who did not receive NAC. It is well known that the effector functions of T cells that express PD-1 in the tumor microenvironment can be downregulated upon activation by PD-L1 (B7-H1) or PD-L2 (B7-DC) [26]. However, certain chemotherapies have been shown to downregulate PD-1 or PD-L1/2 expression [27,28], whereas others increase PD-L1 and PD-1 on tumor cells or peripheral leukocytes from cancer patients [29,30]. This heterogeneity of results can be explained by differences in treatments, techniques, and sources of samples.

Interestingly, we found a positive correlation between the percentages of PD-L1+ neutrophils from urine and matched blood, suggesting a link between urine and circulation. A homologous link between circulation and tumor environment has been suggested previously when cancer-related neutrophils are found simultaneously in circulation and the tumor environment of bladder cancer patients [31].

We found an influence of smoking on urine cellular composition and PD-1 and PD-L1 expression. Along the same line, other authors have described increased total counts of lymphocytes (CD4+ T cells and B cells) and neutrophils in the blood of smokers [32,33] and observed that smoking can upregulate PD-L1/IDO expression through an oxidative stress-dependent mechanism [34]. In addition, smokers had higher IFNγ mRNA and protein levels in gingival tissues compared to non-smokers with comparable types of periodontitis [35]. However, it was demonstrated that IFNγ from lymphocytes induces PD-L1 expression and promotes the progression of cancer [36]. In this line, in a meta-analysis, Jiahang et al. stated that smokers would benefit from either anti-PD-1/PD-L1 or anti-PD-1/PD-L1 combined with chemotherapy [37]. However, one report showed that the expression of PD-1 on the lymphocytes of tumor infiltrates was not associated with the smoking status of lung cancer patients [38], whereas another described that current smokers have significantly lower numbers of CD8+ cytotoxic T cells and PD-L1+ cells than non- and former smokers in head and neck squamous cell carcinoma [39]. Further analysis will be necessary to establish a definitive conclusion regarding the benefits of smoking and to validate the use of urine as a marker for PD-L1 and PD-1 status in smokers.

Although LN affectation influences urine composition, we did not find differences in urine composition when LN+ patients were classified according to NAC or non-NAC. The pathologic stage of LN at the time of RC is the most powerful independent predictor of long-term outcomes [40]. Interestingly, Tian et al. found T2–T4 staging to be negatively correlated with age in LN + MIBC [41]. However, we did not find any age differences between LN+ and LN−. It is unlikely that this apparent contradiction is the result of differences in cohorts because, similar to other groups, we found 20% of patients with LN+ at the time of RC [42].

Bladder cancer is an immune-sensitive tumor and patients with high immune cell infiltration showed the highest cytotoxic scores [43]. Immune infiltrates seem to be indicative of clinical outcomes and drug effectiveness. Particularly, the presence of infiltrating TILs has been associated with a good prognosis in MIBC, and this positive association was modulated by the presence of tumor-associated macrophages [44] and B lymphocytes [45]. Due to this physiological association, it is tempting to speculate that urine composition is related to the bladder tumor microenvironment. We found that the percentage of urine lymphocytes inversely correlated with the average number of infiltrating lymphocytes in matched biopsy specimens. This result suggests that urine lymphocytes came from the tumor because they were not retained in that microenvironment. On the other hand, we were not able to establish a correlation between circulating and infiltrating neutrophils due to the lack of specific markers and the variable amount of neutrophils infiltrating the tumors [46]. However, when identifying and counting CD66+ neutrophils, Takakura et al. were also unable to establish a correlation between neutrophils in biopsies and high NLR in circulation [47]. The presence of different populations of leukocytes in urine may therefore be the result of different mechanisms and may be influenced by genetics and external and internal factors that contribute to infiltrates in the bladder tumor.

With our approach of excluding external factors influencing the composition of urine, we were able to determine that recurring patients with LN− at the time of RC had higher urine lymphocytes, macrophages, and PD-L1+ neutrophils; lower blood NLR and PD-1+ CD8+; and higher PD-L1+ monocytes than patients who had not recurred. This approach to analyzing MIBC patients has been also applied by other authors [22], who found that higher neutrophil infiltration in benign LN may be associated with poorer survival. Our findings were in line with Padmanee et al., who found that the disease-free survival time was shorter for MIBC patients with fewer CD8+ tumor-infiltrating lymphocytes [20]. In other types of cancer, an improved clinical outcome has been associated with the presence of intra-tumoral lymphocytes [48,49].

We are aware that the main limitation of our pilot study was the small number of patients and that our findings should be validated in a larger series. However, our data have opened up the opportunity to use urine cellular composition as a non-invasive tool, not only as an approximation to the tumor environment but also to predict the outcome in groups of patients that are going to be treated with immunotherapy. In this manuscript, the viability of urine leukocytes was also overcome with technical precautions previously applied by other authors [19]. Additionally, our findings reiterate the crucial role of the host immune system in the clinical outcome of MIBC patients and establish a link between urine and tumor circulation. Future experiments to further characterize the phenotype of urine lymphocytes, macrophages, and neutrophils will be necessary.

## Figures and Tables

**Table 1:** Demographic and clinical characteristics of MIBC patients.

	MIBC (*n* = 24)
Age (years, mean ± SD)	67.96 ± 10.58
Male, *n* (%)	18 (75)
Smoking status, *n* (%)	
Yes	15 (62.5)
No	6 (37.5)
Previous treatment to RTU, *n* (%)	
BCG	7 (29.16)
Mitomycin	2 (8.34)
no treatment	15 (62.5)
Neoadjuvant chemotherapy, *n* (%)	
Yes	9 (37.5)
No	15 (62.5)
Lymph node, *n* (%)	
Positive	5 (20.8)
Negative	19 (79.2)
Carcinoma in situ, *n* (%)	
Positive	13 (54.16)
Negative	11 (45.84)

BCG: Bacillus Calmette–Guerin.

**Table 2:** Cell composition of urine and blood from MIBC patients.

	Urine	Blood
%Lymphocytes/CD45+	2.84 (0.79–5.94)	27.91 (20.7–31.22)
%Macrophages-monocytes/CD45+	3.35 (1.34–4.27)	5.06 (3.09–6.97)
%Neutrophils/CD45+	90.75 (80.86–94.95)	62.34 (56.88–67.21)
Neutrophil/Lymphocyte	30.27 (14.22–154.6)	2.13 (1.81–3.38)
Lymphocyte subpopulations	(*n* = 18)	(*n* = 24)
% CD4+	40.24 (23.87–51.24)	52.91 (43.21–58.28)
CD4+PD-L1+/CD4+	0.3 (0–1.33)	0.045(0.01–0.30)
CD4+PD1+/CD4+	20 (3.6–30)	2.03 (1.03–4.91)
%CD8+	19.23 (12–31.08)	23.37 (19.57–32.68)
CD8+PD-L1+/CD8+	3.16 (0–15)	0.79 (0.06–30.87)
CD8+ PD1+/CD8+	15 (0–40)	1.98 (0.65–4.74)
% CD3-CD8+	2.25 (0.92–7.75)	3.77 (1.94–5.5)
% CD3-CD8-	29 (18.78–55.44)	18.83 (16.06–21.24)
%Macrophages-monocytes		
PD-L1+/macrophages	5 (2.68–11.75)	18.85 (13.03–34.98)
PD1+/macrophages	1.39 (0.68–6.18)	0.10 (0–0.59)
%Neutrophils		
PD-L1+/neutrophils	0.51 (0.10–3.64)	16 (3.08–32.18)
PD1+/neutrophils	0.13 (0.03–0.51)	0.02 (0.01–0.09)
Lymphocytes/CD326+	1.48 (0.48–9.02)	
% PD-L1+ CD326+	6.87 (1.87–14.73)	

Number of patients was *n* = 24. The phenotype of the urine lymphocyte subpopulations was determined in MIBC patients with >1% of lymphocytes (*n* = 18). The lymphocyte/CD326+ ratio and the % of PD-L1 + CD326+ cells were determined in 22 patients due to a viability <60% of CD45− cells in two samples. Data are presented as the median of the percentage (IQR: 25%–75% percentile).

## Data Availability

The data presented in this study are available on request from the corresponding author. The data are not publicly available due to ethical restriction.

## Supplementary Materials

The following are available online at https://www.mdpi.com/article/10.3390/biomedicines9091125/s1, Figure S1: Number of viable cells CD45+ and CD326+ for each patient (A); counts of CD45+ per ml and CD326+ per ml of urine for each patient (B) included in the study; Figure S2: Gating hierarchy of a representative sample of urine cells showing how doublets and dead cells were excluded in the flow cytometry analysis. (A) Urine cells were analysed by flow cytometry without Violet dead Cell and without antibodies. (B) Urine were analyzed by flow cytometry with Violet dead Cell and anti-CD45 APC Vio770 and anti-CD326 PE Vio770 antibodies; Figure S3: Phenotyping of (A) urine and (B) blood leukocytes. Gated populations of urine based on viable cells, Lymphocyte population CD4+ and CD8+ based on the expression of CD3 and CD8. Expression of PD-1 and PD-L1 based on the limit detection determined by FMO); Table S1. The composition of urine at the time of RC time is associated with the recurrence of LN (−) in patients.

## References

[1] Ferlay J., Soerjomataram I., Dikshit R., Eser S., Mathers C., Rebelo M., Parkin D.M., Forman D., Bray F. (2015). Cancer incidence and mortality worldwide: Sources, methods and major patterns in GLOBOCAN 2012. Int. J. Cancer.

[2] Witjes J.A., Lebret T., Compérat E.M., Cowan N.C., de Santis M., Bruins H.M., Hernández V., Espinós E.L., Dunn J., Rouanne M. (2017). Updated 2016 EAU Guidelines on Muscle-invasive and Metastatic Bladder Cancer. Eur. Urol..

[3] Witjes J.A., Compérat E., Cowan N.C., de Santis M., Gakis G., Lebret T., Ribal M.J., van der Heijden A.G., Sherif A. (2014). EAU Guidelines on Muscle-invasive and Metastatic Bladder Cancer: Summary of the 2013 Guidelines. Eur. Urol..

[4] Cattaneo F., Motterle G., Zattoni F., Morlacco A., Moro F.D. (2018). The Role of Lymph Node Dissection in the Treatment of Bladder Cancer. Front Surg..

[5] Stein B.J.P., Lieskovsky G., Cote R., Groshen S., Feng A., Boyd S., Skinner E., Bochner B., Thangathurai D., Mikhail M. (2001). Radical Cystectomy in the Treatment of Invasive Bladder Cancer: Long-Term Results in 1 054 Patients. J. Clin. Oncol..

[6] Burger M., Catto J.W.F., Dalbagni G., Grossman H.B., Herr H., Karakiewicz P., Kassouf W., Kiemeney L.A., La Vecchia C., Shariat S. (2013). Epidemiology and risk factors of urothelial bladder cancer. Eur. Urol..

[7] Freedman N.D., Silverman D.T., Hollenbeck A.R., Schatzkin A., Abnet C.C. (2011). Association between smoking and risk of bladder cancer among men and women. JAMA.

[8] Batista R., Vinagre N., Meireles S., Vinagre J., Prazeres H., Leão R., Máximo V., Soares P. (2020). Biomarkers for bladder cancer diagnosis and surveillance: A comprehensive review. Diagnostics.

[9] Vlachostergios P.J., Faltas B.M. (2019). The molecular limitations of biomarker research in bladder cancer. World J. Urol..

[10] Babjuk M., Burger M., Compérat E.M., Gontero P., Mostafid A.H., Palou J., van Rhijn B.W.G., Rouprêt M., Shariat S.F., Sylvester R. (2019). European Association of Urology Guidelines on Non-muscle-invasive Bladder Cancer (TaT1 and Carcinoma In Situ) 2019 Update. Eur. Urol..

[11] Prasad S., Tyagi A.K., Aggarwal B.B. (2016). Minireview Detection of inflammatory biomarkers in saliva and urine: Potential in diagnosis, prevention, and treatment for chronic diseases. Exp. Biol. Med..

[12] De Boer E.C., de Jong W.H., van der Meijden A.P.M., Steerenberg P.A., Witjes F., Vegt P.D.J., Debruyne F.M.J., Ruitenberg E.J. (1991). Leukocytes in the urine after intravesical BCG treatment for superficial bladder cancer—A flow cytofluorometric analysis. Urol. Res..

[13] Black A.J., Sc B., Zargar H., Zargar-shoshtari K., Fairey A.S., Mertens L.S., Dinney C.P., Mir M.C., Krabbe L.M., Cookson M.S. (2020). The prognostic value of the neutrophil-to-lymphocyte ratio in patients with muscle-invasive bladder cancer treated with neoadjuvant chemotherapy and radical cystectomy. Urol. Oncol. Semin. Orig. Investig..

[14] Guillamo C.F., Gimeno L., Server G., Martínez-Sánchez M.V., Escudero J.F., López-Cubillana P., Cabezas-Herrera J., Campillo J.A., Abellan D.J., Martínez-García J. (2021). Immunological Risk Stratification of Bladder Cancer Based on Peripheral Blood Natural Killer Cell Biomarkers. Eur. Urol. Oncol..

[15] Shaul M.E., Fridlender Z.G. (2018). Cancer-related circulating and tumor-associated neutrophils—Subtypes, sources and function. FEBS J..

[16] Pfistershammer K., Majdic O., Stöckl J., Zlabinger G., Kirchberger S., Steinberger P., Knapp W. (2004). CD63 as an Activation-Linked T Cell Costimulatory Element. J. Immunol..

[17] Wong Y.N.S., Joshi K., Khetrapal P., Ismail M., Reading J.L., Sunderland M.W., Georgiou A., Furness A.J.S., Ben Aissa A., Ghorani E. (2018). Urine-derived lymphocytes as a non-invasive measure of the bladder tumor immune microenvironment. J. Exp. Med..

[18] Witjes J.A., Bruins H.M., Cathomas R., Compérat E.M., Cowan N.C., Gakis G., Hernández V., Linares Espinós E., Lorch A., Neuzillet Y. (2020). European Association of Urology Guidelines on Muscle-invasive and Metastatic Bladder Cancer: Summary of the 2020 Guidelines. Eur. Urol..

[19] Sakatsume M., Xie Y., Ueno M., Obayashi H., Goto S., Narita I., Homma N., Tasaki K., Suzuki Y., Gejyo F. (2001). Human glomerulonephritis accompanied by active cellular infiltrates shows effector T cells in urine. J. Am. Soc. Nephrol..

[20] Sharma P., Shen Y., Wen S., Yamada S., Jungbluth A.A., Gnjatic S., Bajorin D.F., Reuter V.E., Herr H., Old L.J. (2007). CD8 tumor-infiltrating lymphocytes are predictive of survival in muscle-invasive urothelial carcinoma. Proc. Natl. Acad. Sci. USA.

[21] Seiler R., Gibb E.A., Wang N.Q., Oo H.Z., Lam H.M., van Kessel K.E., Voskuilen C.S., Winters B., Erho N., Takhar M.M. (2019). Divergent biological response to neoadjuvant chemotherapy in muscle-invasive bladder cancer. Clin. Cancer Res..

[22] Pal S.K., Pham A., Vuong W., Liu X., Lin Y., Ruel N., Yuh B.E., Chan K., Wilson T., Lerner S.P. (2017). Prognostic Significance of Neutrophilic Infiltration in Benign Lymph Nodes in Patients with Muscle-invasive Bladder Cancer. Eur. Urol. Focus.

[23] Suttmann H., Riemensberger J., Bentien G., Schmaltz D., Stöckle M., Jocham D., Böhle A., Brandau S. (2006). Neutrophil granulocytes are required for effective Bacillus Calmette-Guérin immunotherapy of bladder cancer and orchestrate local immune responses. Cancer Res..

[24] De Franco J.E. (2010). Neutrophils: Lifespan, Functions and Roles in Disease.

[25] Wang T.-T., Zhao Y.-L., Peng L.-S., Chen N., Chen W., Lv Y.-P., Mao F.-Y., Zhang J.-Y., Cheng P., Teng Y.-S. (2017). Tumour-activated neutrophils in gastric cancer foster immune suppression and disease progression through GM-CSF-PD-L1 pathway. Gut.

[26] Keir M.E., Butte M.J., Freeman G.J., Sharpe A.H. (2008). PD-1 and its ligands in tolerance and immunity. Annu. Rev. Immunol..

[27] Oki Y., Buglio D., Zhang J., Ying Y., Zhou S., Sureda A., Ben-Yehuda D., Zinzani P.L., Prince H.M., Harrison S.J. (2014). Immune regulatory effects of panobinostat in patients with Hodgkin lymphoma through modulation of serum cytokine levels and T-cell PD1 expression. Blood Cancer J..

[28] Sherif A., Winerdal M., Winqvist O. (2018). Immune Responses to Neoadjuvant Chemotherapy in Muscle Invasive Bladder Cancer. Bladder Cancer.

[29] Shin J., Chung J.H., Kim S.H., Lee K.S., Suh K.J., Lee J.Y., Kim J.W., Lee J.O., Kim J.W., Kim Y.J. (2019). Effect of platinum-based chemotherapy on PD-L1 expression on tumor cells in non-small cell lung cancer. Cancer Res. Treat.

[30] Peng J., Hamanishi J., Matsumura N., Abiko K., Murat K., Baba T., Yamaguchi K., Horikawa N., Hosoe Y., Murphy S.K. (2015). Chemotherapy induces programmed cell death-ligand 1 overexpression via the nuclear factor-κBto foster an immunosuppressive tumor microenvironment in Ovarian Cancer. Cancer Res..

[31] Eruslanov E., Neuberger M., Daurkin I., Perrin G.Q., Algood C., Dahm P., Rosser C., Vieweg J., Gilbert S.M., Kusmartsev S. (2012). Circulating and tumor-infiltrating myeloid cell subsets in patients with bladder cancer. Int. J. Cancer.

[32] Pedersen K.M., Çolak Y., Ellervik C., Hasselbalch H.C., Bojesen S.E., Nordestgaard B.G. (2019). Smoking and Increased White and Red Blood Cells: A Mendelian Randomization Approach in the Copenhagen General Population Study. Arterioscler. Thromb. Vasc. Biol..

[33] Andreoli C., Bassi A., Gregg E.O., Nunziata A., Puntoni R., Corsini E. (2015). Effects of cigarette smoking on circulating leukocytes and plasma cytokines in monozygotic twins. Clin. Chem. Lab. Med..

[34] Kerdidani D., Magkouta S., Chouvardas P., Karavana V., Glynos K., Roumelioti F., Zakynthinos S., Wauters E., Janssens W., Lambrechts D. (2018). Cigarette Smoke–Induced Emphysema Exhausts Early Cytotoxic CD8 + T Cell Responses against Nascent Lung Cancer Cells. J. Immunol..

[35] César-Neto J.B., Duarte P.M., De Oliveira M.C.G., Casati M.Z., Tambeli C.H., Parada C.A., Sallum E.A., Nociti F.H. (2006). Smoking modulates interferon-γ expression in the gingival tissue of patients with chronic periodontitis. Eur. J. Oral Sci..

[36] Abiko K., Matsumura N., Hamanishi J., Horikawa N., Murakami R., Yamaguchi K., Yoshioka Y., Baba T., Konishi I., Mandai M. (2015). IFN-γ from lymphocytes induces PD-L1 expression and promotes progression of ovarian cancer. Br. J. Cancer.

[37] Mo J., Hu X., Gu L., Chen B., Khadaroo P.A., Shen Z., Dong L., Lv Y., Chitumba M.N., Liu J. (2020). Smokers or non-smokers: Who benefits more from immune checkpoint inhibitors in treatment of malignancies? An up-to-date meta-analysis. World J. Surg. Oncol..

[38] Calles A., Liao X., Sholl L.M., Rodig S.J., Freeman G.J., Butaney M., Lydon C., Dahlberg S.E., Hodi F.S., Oxnard G.R. (2015). Expression of PD-1 and Its Ligands, PD-L1 and PD-L2, in Smokers and Never Smokers with KRAS-Mutant Lung Cancer. J. Thorac. Oncol..

[39] Chung C.H., de la Iglesia J.V., Wang X., Song F., Chaudhary R., Masannat J., Conejo-Garcia J., Hernandez-Prera J., Slebos R. (2019). Tobacco smoking is associated with the immune suppressive microenvironment in head and neck squamous cell carcinoma (HNSCC). Ann. Oncol..

[40] Hautmann R.E., De Petriconi R.C., Pfeiffer C., Volkmer B.G. (2012). Radical cystectomy for urothelial carcinoma of the bladder without neoadjuvant or adjuvant therapy: Long-term results in 1100 patients. Eur. Urol..

[41] Tian Z., Meng L., Wang X., Diao T., Hu M., Wang M., Liu M., Wang J. (2020). Young age increases the risk of lymph-node metastasis in patients with muscle-invasive bladder urothelial carcinoma. BMC Cancer.

[42] Li F., Hong X., Hou L., Lin F., Chen P., Pang S., Du Y., Huang H., Tan W. (2016). A greater number of dissected lymph nodes is associated with more favorable outcomes in bladder cancer treated by radical cystectomy: A meta-analysis. Oncotarget.

[43] Pan S., Zhan Y., Chen X., Wu B., Liu B. (2019). Bladder cancer exhibiting high immune infiltration shows the lowest response rate to immune checkpoint inhibitors. Front. Oncol..

[44] Sjödahl G., Lövgren K., Lauss M., Chebil G., Patschan O., Gudjonsson S., Månsson W., Fernö M., Leandersson K., Lindgren D. (2014). Infiltration of CD3+ and CD68+ cells in bladder cancer is subtype specific and affects the outcome of patients with muscle-invasive tumors. Urol. Oncol..

[45] Mahmoud S.M.A., Lee A.H.S., Paish E.C., MacMillan R.D., Ellis I.O., Green A.R. (2012). The prognostic significance of B lymphocytes in invasive carcinoma of the breast. Breast Cancer Res. Treat..

[46] Shaul M.E., Fridlender Z.G. (2019). Tumour-associated neutrophils in patients with cancer. Nat. Rev. Clin. Oncol..

[47] Takakura K., Ito Z., Suka M., Kanai T., Matsumoto Y., Odahara S., Matsudaira H., Haruki K., Fujiwara Y., Saito R. (2016). Comprehensive assessment of the prognosis of pancreatic cancer: Peripheral blood neutrophil-lymphocyte ratio and immunohistochemical analyses of the tumour site. Scand. J. Gastroenterol..

[48] Sato E., Olson S.H., Ahn J., Bundy B., Nishikawa H., Qian F., Jungbluth A.A., Frosina D., Gnjatic S., Ambrosone C. (2005). Intraepithelial CD8+ tumor-infiltrating lymphocytes and a high CD8+/regulatory T cell ratio are associated with favorable prognosis in ovarian cancer. Proc. Natl. Acad. Sci. USA.

[49] Nakano O., Naito Y., Nagura H., Ohtani H., Nakano O., Sato M., Suzuki K., Orikasa S., Aizawa M., Suzuki Y. (2001). Proliferative activity of intratumoral CD8+ T-lymphocytes as a prognostic factor in human renal cell carcinoma: Clinicopath-ologic demonstration of antitumor immunity. Cancer Res..

