# Supplementary material for: Immunological Status of Bladder Cancer Patients Based on Urine Leukocyte Composition at Radical Cystectomy
Source: Biomedicines. 2021 Aug 31;9(9):1125. doi: 10.3390/biomedicines9091125 (PMC8465292; doi:10.3390/biomedicines9091125)
Supplement: Supplementary file 1 [file biomedicines-09-01125-s001.zip › biomedicines-1331870-supplementary (1).pdf]

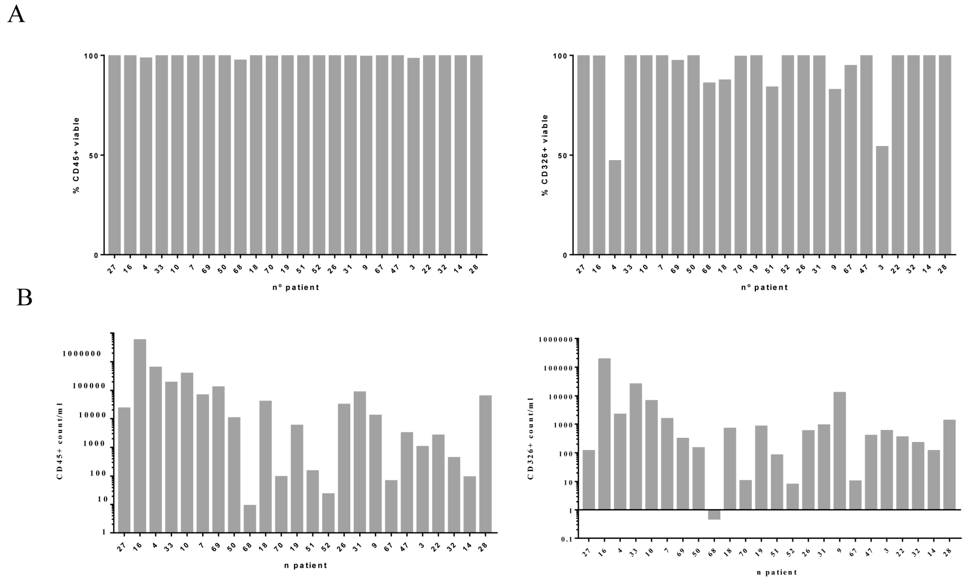

Supplementary Figure S1. Number of viable cells CD45+ and CD326+ for each patient (A); counts of CD45+ per ml and CD326+ per ml of urine for each patient (B) included in the study.

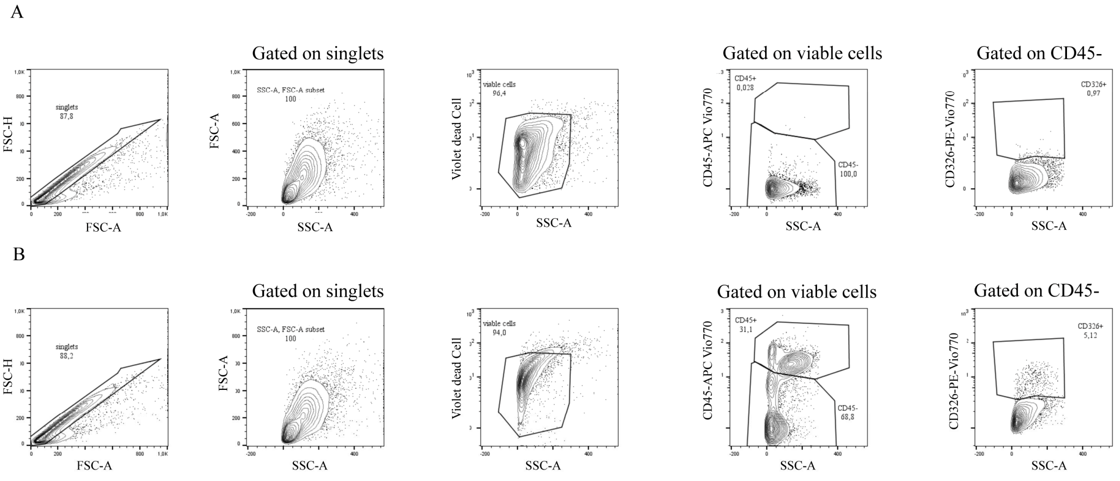

Supplementary Figure S2. Gating hierarchy of a representative sample of urine cells showing how doublets and dead cells were excluded in the flow cytometry analysis. (A) Urine cells were analysed by flow cytometry without Violet dead Cell and without antibodies. (B) Urine were analyzed by flow cytometry with Violet dead Cell and anti-CD45 APC Vio770 and anti-CD326 PE Vio770 antibodies).

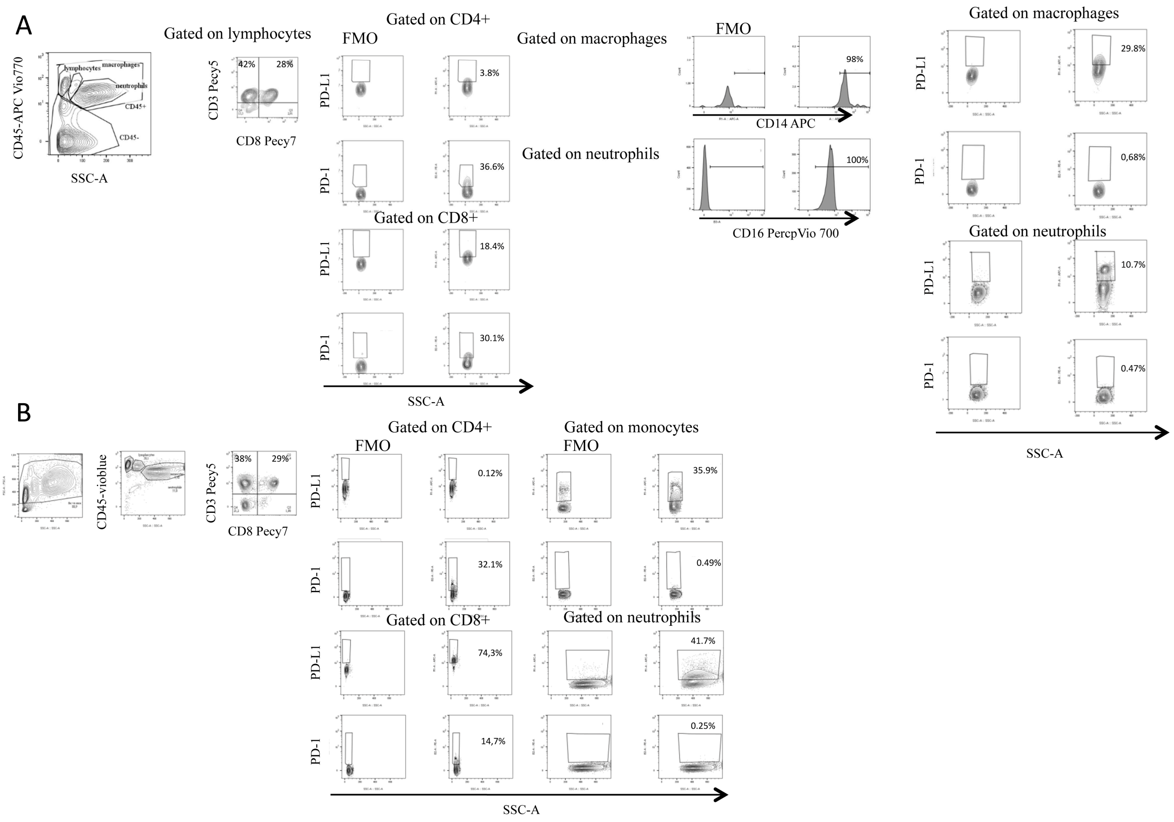

Supplementary Figure S3. Phenotyping of (A) urine and (B) blood leukocytes. Gated populations of urine based on viable cells, Lymphocyte population CD4+ and CD8+ based on the expression of CD3 and CD8. Ex-pression of PD-1 and PD-L1 based on the limit detection determined by FMO).

Supplementary Table S1. The composition of urine at the time of RC time is associated with the recurrence of LN (-) in patients.

	**Urine**			Blood		
	**Recurrence**	No recurrence	*P*	Recurrence	No recurrence	*P*
% lymphocytes	2.3 (1.2–6.3)	0.4 (0.1–1.6)		22.6 (16.1–28.0)	30.9 (24.8–34.3)	
% macrophages	3.4 (2.3–3.9)	1.2 (0.6–1.5)	0.01	3.1 (2.1–6.6)	6.2 (3.07–7.7)	
% neutrophils	91.2 (88.4–94.9)	96.6 (93–98)	0.03	67 (64.1–74.0)	57.6 (52.6–61.9)	
NLR	39.7 (13.7–114.7)	213 (57.7–515.8)		3.02 (2.3–4.6)	1.8 (1.4–2.4)	
%PDL-1+						
macrophages	5.1 (2.1–11.4)	3.6 (1.1–6.7)		30.3 (20.08–67.5)	13.1 (10–17.7)	0.03
neutrophils	0.5 (0.4–6.5)	0.07 (0.04–0.3)	0.02	11.2 (2.4–33.4)	3.6 (3–14)	
%PD-1+						
macrophages	2.2 (1.2–4.1)	1.11 (0.8–4.1)		0.14 (0.03–0.3)	0.6 (0–0.8)	
neutrophils	0.1 (0.03–0.5)	0.04 (0–0.7)		0.01 (0.01–0.05)	0.02 (0.01–0.09)	
CD4+				1.2 (1.09–3.1)	4.8 (1.2–10.2)	
CD8+				0.5 (0.2–2.1)	4.9 (1.6–9.5)	0.03
CD3-CD8+				0.1 (0–0.5)	0.2 (0–1.6)	
CD3-CD8-				0.2 (0.02–0.4)	0.1 (0.1–1.4)	
